# Pain perception and attitudes toward life-sustaining treatment in pediatric patients with disorders of consciousness: a survey of Chinese healthcare professionals

**DOI:** 10.3389/fneur.2026.1765041

**Published:** 2026-07-06

**Authors:** Chunyan Yang, Meiqi Li, Yufei Xue, Fangting Wang, Wangshan Huang, Xiaochen Liu, Xiangyue Xiao, Xuhang Fan, Feng Chen, Steven Laureys, Haibo Di, Siyu Dai

**Affiliations:** 1 International Institute for Vegetative State and Consciousness Science, Hangzhou Normal University, Hangzhou, China; 2 Zhejiang-Belgium Joint Laboratory for Disorders of Consciousness, Hangzhou Normal University, Hangzhou, China; 3 School of Public Health and Nursing, Hangzhou Normal University, Hangzhou, China; 4 Hangzhou First People’s Hospital, Hangzhou, China; 5 Brain Hospital, Tongji University, Shanghai, China; 6 School of Nursing, The University of Hong Kong, Pok Fu Lam, Hong Kong SAR, China; 7 International Associated Laboratory of Consciousness Research, CERVO Brain Research Center, Université Laval, Québec City, QC, Canada; 8 School of Clinical Medicine, Hangzhou Normal University, Hangzhou, China; 9 Faculty of Medicine, The Chinese University of Hong Kong, Pok Fu Lam, Hong Kong SAR, China

**Keywords:** decision-making, disorders of consciousness, ethics, healthcare professionals, life-sustaining treatment, pain, pediatric patients

## Abstract

**Background:**

Managing pain and life-sustaining treatment (LST) for pediatric patients with disorders of consciousness (DoC) presents unique neuroethical dilemmas. This study investigated the attitudes of Chinese healthcare professionals (HCPs) toward pain perception and LST in pediatric DoC, specifically exploring the influence of decision-making perspectives and socioeconomic factors.

**Methods:**

A cross-sectional survey was conducted among 656 Chinese HCPs (March–November 2025). Participants evaluated LST limitation and pain perception for unresponsive wakefulness syndrome (UWS) and minimally conscious state (MCS) from third-person (for a patient) and first-person (for their own child) perspectives. Multivariate logistic regression identified predictors.

**Results:**

HCPs were more supportive of limiting LST for UWS (52.1%) than MCS (31.6%) in third-person contexts (*p* < 0.001). In first-person scenarios, support dropped to 34.0% (UWS) and 18.7% (MCS). High income (>20,000 CNY) predicted LST limitation for UWS in third-person context (AOR = 2.87). In first-person scenarios, nursing (AOR = 0.46) and rehabilitation therapists (AOR = 0.55) were less likely to continue LST for their own child with UWS, while childlessness was associated with greater willingness to continue (AOR = 2.14). Pain perception was endorsed for MCS (89.9%) and UWS (65.1%), and significantly reduced willingness to limit LST in UWS cases, particularly from a first-person perspective (*p* = 0.002).

**Conclusion:**

Chinese HCPs are cautious about limiting LST in pediatric DoC, with attitudes shaped by diagnosis, kinship perspective, and economic context. Widespread recognition of pain perception in non-communicative children underscores a critical ethical boundary. A culturally sensitive, evidence-based decision-support framework integrating neuroplasticity, socioeconomic reality, and pain ethics is needed.

## 1 Introduction

Disorders of consciousness (DoC) are a neurological crisis caused by severe brain injury, with subtypes including the vegetative state (VS)/unresponsive wakefulness syndrome (UWS) and the minimally conscious state (MCS) (1–4). VS/UWS refers to patients who show wakefulness without behavioral evidence of awareness, with no voluntary behavior or communication capacity; MCS refers to patients who have partial consciousness and can sporadically exhibit voluntary behavior and limited communication (1). These patients not only completely or partially lose perception of self and environment but also often depend on long-term life-sustaining treatment (LST), posing a heavy burden on the healthcare system, families, and society.

Compared to adults, medical decision-making for pediatric and adolescent DoC patients is more complex due to the unique properties of the developing central nervous system, their inability to express themselves, and the distinct nature of family emotional bonds (5–9). This complexity presents a dual ethical challenge. First, while the rehabilitation potential of children with DoC is highly uncertain (8, 10–12), their developing brains possess greater neural plasticity and capacity for functional reorganization (10, 13). Consequently, decisions regarding LST involve a value-based dilemma, weighing the probability of survival against potential neurological functional outcomes (14–16). Some studies suggest that younger patients may demonstrate better potential for consciousness recovery and functional outcomes than adults (17, 18). For instance, Eilander et al. (17) found that in pediatric traumatic DoC patients, the functional connectivity remodeling of the default mode network was significantly correlated with behavioral improvements. Simultaneously, assessing consciousness and pain in this population poses distinct challenges. Widely used behavioral scales such as the CRS-R are primarily validated in adult populations, and there is a current lack of objective methods to evaluate pain perception in children with DoC. Clinical assessments primarily rely on indirect behavioral indicators to infer their subjective experiences (19, 20). Furthermore, from a life-course perspective, these children face not only the maintenance of current life but also the potential loss of their entire developmental future. This profound ethical weight further complicates LST decisions (21, 22).

An attitude is a relatively stable psychological tendency formed by an individual toward a specific object. It contains three core components: cognitive evaluation, affective response, and behavioral intention, and is expressed as a directional (positive or negative) evaluative response (23). In medical decision-making, this process requires healthcare professionals (HCPs) to seek an ethical balance in specific clinical contexts, that is, to seek a dynamic equilibrium between respecting the patient’s potential autonomy and protecting their vulnerability (24). The attitudes of HCPs affect the surrogate decision-making process of family members and play a key role in ensuring that treatment plans conform to the patient’s/family’s values (25, 26). It is noteworthy that in decisions involving LST, the difference in decision perspective—namely, “deciding for another’s child” (third-person) versus “deciding for one’s own child” (first-person)—may lead to significantly different ethical judgments. Identifying and measuring this perspective difference holds important implications for understanding real-world clinical ethical decision-making (3, 27, 28). Previous research results show that a third-person perspective often more readily leads to theoretical judgments favoring treatment limitation, whereas when reporting subjectively from a first-person perspective, decision-makers need to undergo deeper ethical transposition and emotional engagement (27, 28). A study by Muriel et al. found that because doctors and nurses have extensive contact with hospitals and patients, they were less likely to wish for active treatment if they themselves were in a persistent vegetative state.

In addition, some studies have found that pain is not only a source of suffering for patients but has also become a key factor in determining LST strategies (29, 30), An area that is under-researched in the field of disorder of consciousness.

In addition to attitudes and perspective, other factors influencing the LST decision-making process include professional, personal, and cultural beliefs, as well as social factors. The combined effect of these factors results in significant differences in LST practices between different centers and regions, which in turn leads to a post-LST mortality rate ranging from 40 to 90% in both adults and children (31–33). At present, research evidence on children with DoC is relatively scarce, and existing studies often neglect the influence of the particularities of child neurodevelopment on decision-making (5). However, existing research has predominantly been conducted in Western contexts, and its applicability to non-Western settings remains to be fully examined. In these non-Western settings, healthcare professionals’ (HCP) attitudes generally tend toward limiting life-sustaining treatment (LST) (3, 34–36). Related research in China is in a preliminary stage, lacking ethical guidance that fits the national context. Therefore, we hypothesize that there are significant differences between the survey results on limiting LST for children versus adults with DoC (36).

This study sets out to survey Chinese HCPs’ attitudes on pain perception and LST for children with DoC and to examine the underlying influencing factors. Specifically, it focuses on how the decision perspective (treating another’s child versus one’s own child) shapes these attitudes, thereby aiming to provide empirical evidence for building a clinically and culturally congruent neuroethics decision-support framework for children in China.

## 2 Methods

### 2.1 Study design

This study employed a cross-sectional survey using a questionnaire adapted from a previously published study by the Coma Science Group. The original instrument focused on significant differences in healthcare professionals’ attitudes toward life-sustaining treatments for adult patients with disorders of consciousness. The cultural adaptation procedures were as follows: the English questionnaire was translated into Chinese using a back-translation method. The forward translation was performed by a native Chinese-speaking bilingual researcher, and the back translation was conducted independently by another bilingual researcher who had no prior access to the original version. The original English version and the back-translated version were compared by a reviewer with relevant research background to assess semantic, conceptual, and cultural equivalence. Subsequently, an expert panel consisting of three researchers in the field of disorders of consciousness pretested the draft translation, discussed any ambiguous or inconsistent items item-by-item, and revised the questionnaire according to expert feedback (37, 38). The adapted version consists of three sections: demographic characteristics of healthcare professionals (including age, gender, education level, occupation, and religious beliefs), their perceptions of pain in pediatric patients with DoC, and their attitudes toward life-sustaining treatment for these children.

### 2.2 Study participants and data collection

A convenience sampling method was employed to recruit participants at academic conferences on disorders of consciousness held in eight Chinese cities (including Shanghai, Hangzhou, Beijing, etc.) between March 2025 to November 2025. The target population included: specialized healthcare professionals in disorders of consciousness (such as clinicians and therapists from neurology, neurosurgery, critical care, traditional Chinese medicine rehabilitation, and rehabilitation medicine departments), as well as medical and related professionals (doctors from other departments). Inclusion criteria were: ① age ≥18 years; ② normal communication ability; ③ Chinese nationality and native-born (without immigration background) to ensure homogeneity in cultural background and cognitive characteristics among the study subjects. The questionnaire was administered in both paper and electronic formats: paper versions were distributed on-site by researchers during conferences, collected immediately, and checked for completeness (questionnaires with >5% missing items were considered invalid); electronic versions were accessed via encrypted QR codes generated through the Wenjuanxing platform displayed on conference boards, which participants scanned to complete and submit on-site, with data being retrieved promptly by researchers.

The target sample size was determined based on conventional guidelines for survey-based research. Following the rule of thumb for multivariable logistic regression analyses (at least 10 events per predictor variable) and considering an anticipated dropout rate, we aimed to recruit a minimum of 500 participants to ensure adequate statistical power for identifying factors associated with LST attitudes (39, 40). This sample size also exceeds the general recommendation for cross-sectional studies to yield stable estimates of population parameters.

### 2.3 Statistical analysis

This study utilized SPSS 25.0 for statistical analysis. Descriptive statistics were employed to summarize demographic data, with categorical variables presented as frequencies (percentages) and continuous variables as mean ± standard deviation. The chi-square test was used to analyze differences between categorical variables, such as demographic characteristics and other categorical outcomes (e.g., LST: favor limitation/oppose limitation, willing to continue/unwilling to continue; pain perception: yes/no). Bonferroni correction was applied in post-hoc pairwise comparisons. Multivariable logistic regression analysis was conducted to identify potential factors influencing healthcare professionals’ attitudes toward LST, including age, education level, monthly income, specific occupation, and whether they have children. The significance level was set at 0.05 (two-tailed).

Regarding missing data, questionnaires with more than 5% missing items were excluded from the analysis. For the remaining questionnaires, missing values in key variables were minimal (<1%) and handled by pairwise deletion in the respective analyses, as the pattern of missingness was assumed to be completely at random.

## 3 Results

**Figure 1:**
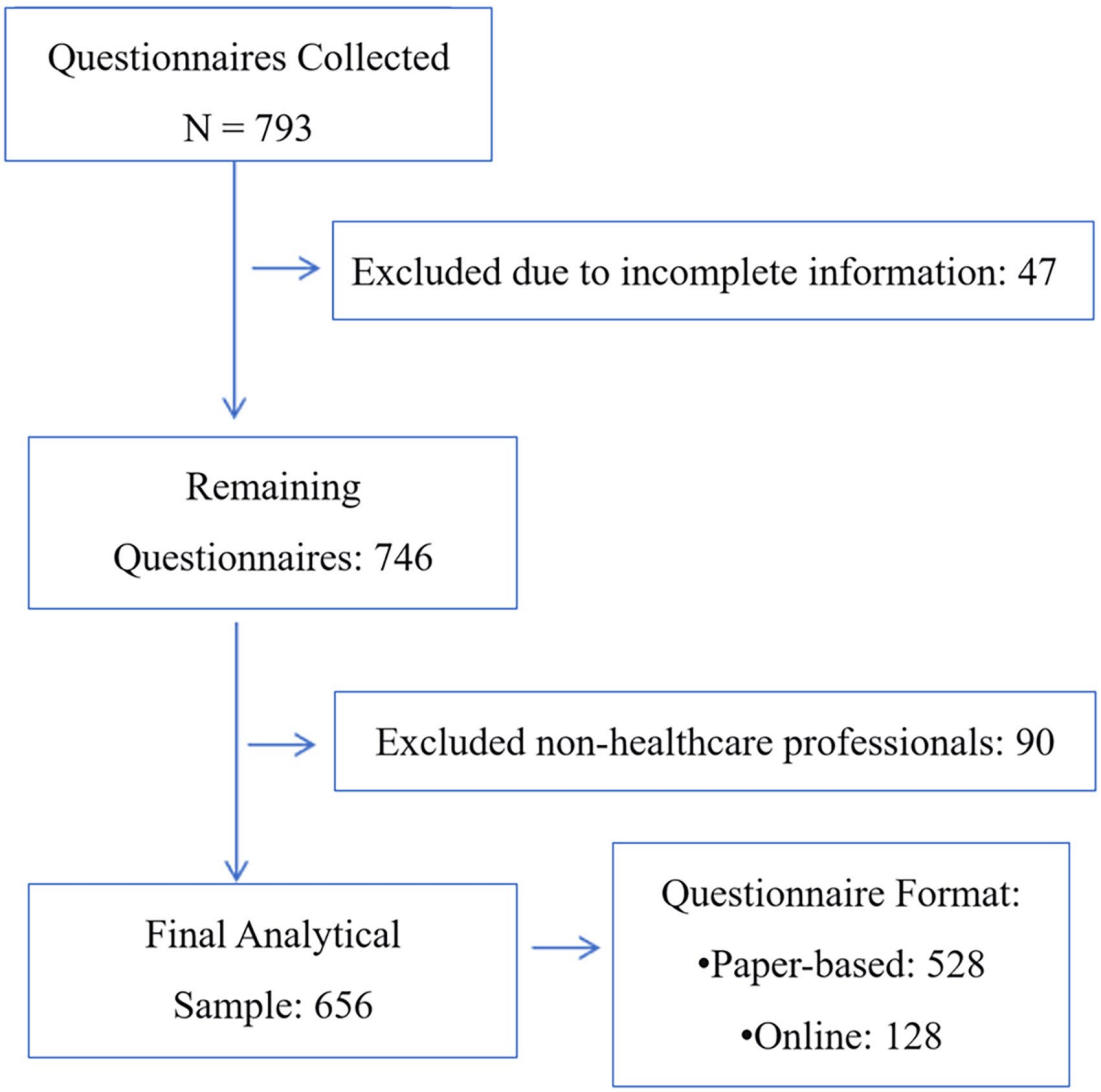
Flow diagram of study questionnaire collection and screening.

A total of 793 questionnaires were initially collected for this study. Following the exclusion of 47 questionnaires (32 paper-based with incomplete demographic information and 15 online with completion times under 40 s) and the further removal of 90 responses from non-healthcare professionals, 656 healthcare professionals (HCPs) were ultimately included in the subsequent analysis (as shown in Figure 1). The participants were from eight provinces/municipalities in China—Beijing (32, 6%), Hangzhou (150, 28%), Shanghai (46, 8.7%), Anhui (57, 10.7%), Hunan (67, 12.6%), Shanxi (54, 10.2%), Zhuhai (44, 8.3%), and Shandong (52, 9.8%)—with the sample heavily concentrated in major, economically developed urban areas with abundant medical resources. While the paper-based questionnaires (528, 80.5%) achieved a 100% response rate, an overall response rate could not be calculated for the online questionnaires (128, 19.5%) due to the use of an electronic distribution link.

### 3.1 Demographic characteristics of healthcare professionals

The demographic characteristics of the participants are summarized in Table 1. Among the 656 respondents, 65.1% were female. The vast majority reported no religious affiliation (90.2%), while 4.3% identified as Buddhist. In terms of monthly income, 45.1% fell within the range of 5,000–10,000 CNY. Most healthcare professionals had received higher education (56.3%). Occupational distribution was as follows: neurologists (20.7%), other clinical physicians (19.5%), nursing staff (28.0%), rehabilitation therapists/technicians (14.9%), and other healthcare personnel (16.7%). Additionally, more than half of the participants had children (*n* = 352, 53.7%).

**Table 1:** Demographic characteristics of the study sample.

Item	Category	*n* (%)
Age	20–30	294 (44.8%)
	31–40	256 (39.0%)
	41–50	77 (11.7%)
	>50	29 (4.4%)
Gender	Male	229 (34.9%)
	Female	427 (65.1%)
Religion	Buddhist	28 (4.3%)
	Christian	3 (0.5%)
	Muslim	2 (0.3%)
	Other	5 (0.8%)
	None	592 (90.2%)
	Missing	26 (3.9%)
Monthly income (CNY)	≤2,000	66 (10.1%)
	2,001–5,000	71 (10.8%)
	5,001-10,000	296 (45.1%)
	10,001–20,000	184 (28.0%)
	>20,000	39 (5.9%)
Education level	Bachelor’s degree	375 (57.2%)
	Master’s degree	281 (42.8%)
Professional category	Neurologist	136 (20.7%)
	Other clinical doctor	128 (19.5%)
	Nursing staff	184 (28.0%)
	Rehabilitation therapist	98 (14.9%)
	Other healthcare professionals	110 (16.7%)
Number of children	None	304 (46.3%)
	One	210 (32.0%)
	Two or more	152 (23.2%)

### 3.2 Perspectives on limiting life-sustaining treatment for pediatric patients with disorders of consciousness

**Figure 2:**
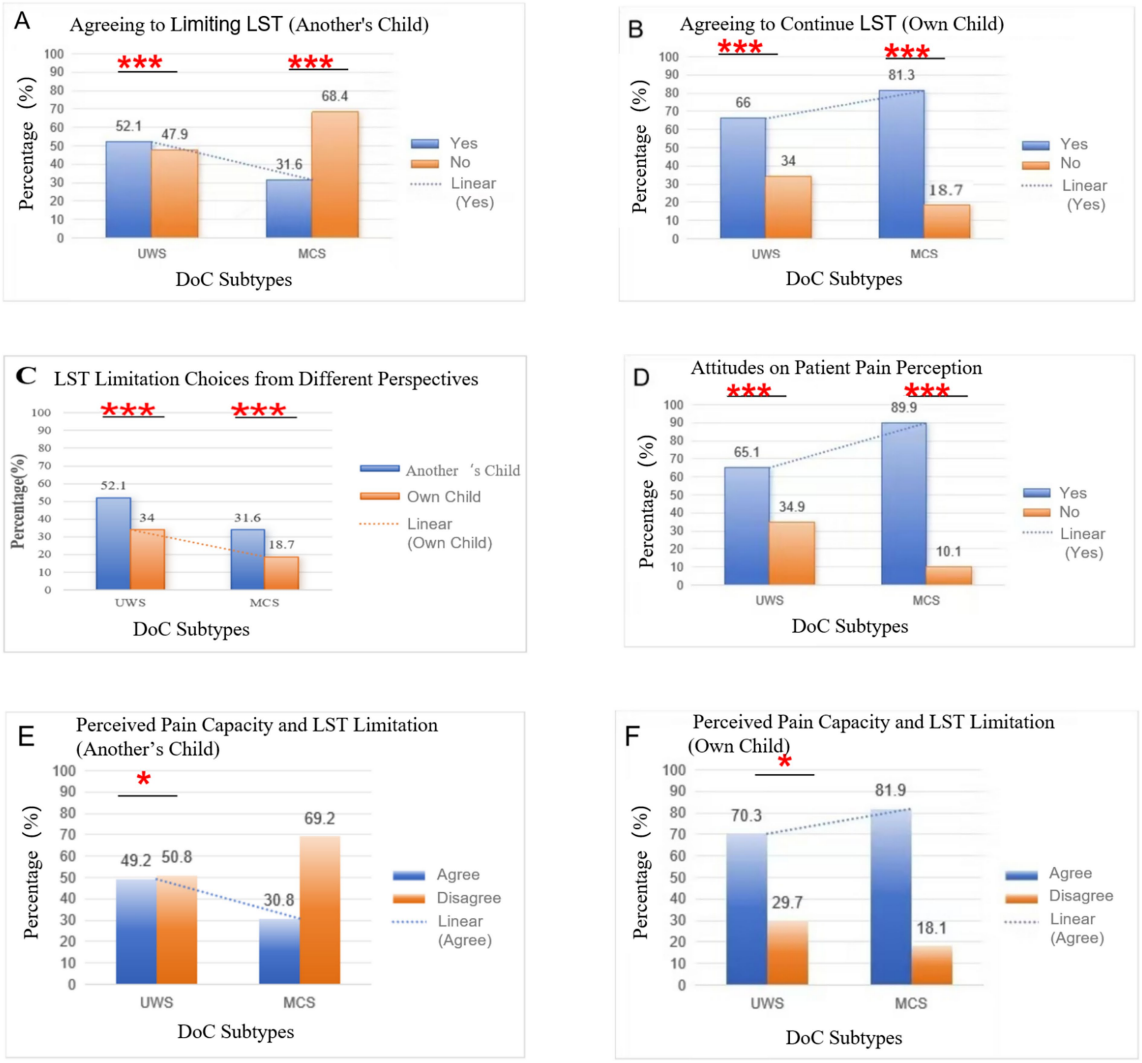
Analysis of participants’ multidimensional attitudes toward LST decision-making for pediatric DoC patients. **(A)** Agreeing to Limiting LST; **(B)** Agreeing to continue LST; **(C)** LST choices from different perspectives; **(D)** Attitudes on patient pain perception; **(E)** Influence of perceived pain capacity on LST limitation (another’s child); **(F)** Influence of perceived pain capacity on LST limitation (own child). “Perceived pain capacity” refers to the belief that the pediatric patient can feel pain. *p < 0.05, ***p < 0.001.

Healthcare professionals’ attitudes toward limiting life-sustaining treatment varied significantly based on the disorder of consciousness (DoC) diagnosis in pediatric patients (Figure 2A). The proportion of respondents who supported limiting LST was higher for patients with unresponsive wakefulness syndrome (UWS) (52.1%) than for those in a minimally conscious state (MCS) (31.6%). A significant difference was observed between these groups following chi-square testing (Figure 2A), suggesting that attitudes toward LST varied by the level of consciousness. In the multivariable logistic regression analysis, monthly income >20,000 CNY was associated with approving LST limitation for UWS (AOR = 2.87, 95% CI: 1.19–6.88, *p* < 0.05), but not for MCS (Table 2).

**Table 2:** Factors associated with HCPs’ attitudes toward LST for pediatric patients with DoC (*n* = 656).

Item	Approve limiting LST for UWS			Approve limiting LST for MCS		
	Yes	No	AOR (95% CI)	Yes	No	AOR (95% CI)
Income >20,000	16 (41.0%)	23 (59.0%)	2.87 (1.19–6.88)*	12 (30.8%)	27 (69.2%)	0.73 (0.28–1.88)

AOR, adjusted odds ratio; CI, confidence interval; CNY, Chinese Yuan. *p < 0.05. Reference categories for all variables are specified in the supplementary tables (Supplementary Tables 1–6).

When participants were asked whether they would continue LST if their own child were a DoC patient (Figure 2B), Chi-square analysis revealed that 66.0% would continue LST for a child with UWS, while this proportion increased significantly to 81.3% if the child were in MCS (*χ*^2^ = 225.992, *p* < 0.001). These proportions were significantly higher than the rates of supporting LST limitation for another person’s child (third-person perspective).

After Bonferroni correction, several factors, including age (*χ*^2^ = 8.885, *p* = 0.031), education level (*χ*^2^ = 9.472, *p* = 0.002), specific occupation (*χ*^2^ = 21.159, *p* = 0.001), and number of children (*χ*^2^ = 10.266, *p* = 0.006), were significantly associated with attitudes toward limiting LST for one’s own child with UWS.

Binary logistic regression (Table 3) identified key predictors of HCPs’ willingness to continue LST for their own child with DoC. When the scenario involved their own child, professional background emerged as a significant determinant. Compared to neurologists (the reference group), nursing staff (AOR = 0.46, 95% CI: 0.26–0.81) and rehabilitation therapists (AOR = 0.55, 95% CI: 0.30–0.99) were significantly less likely to favor continuing LST for a child with UWS. A similar trend was observed for MCS patients among rehabilitation therapists (AOR = 0.40, 95% CI: 0.18–0.88). Additionally, HCPs with no children (AOR = 2.14, 95% CI: 1.28–3.57) demonstrated a significantly higher inclination to continue LST for a UWS child compared to those who were already parents. Regarding income, a monthly income of 10,001–20,000 CNY was associated with a greater willingness to continue LST for an MCS child (AOR = 2.51, 95% CI: 1.07–5.90) from the first-person perspective.

**Table 3:** Predictors of HCPs’ willingness to continue LST for their own child with DoC (*n* = 656).

Item	Approve continue LST for UWS			Approve continue LST for MCS		
	Yes	No	AOR (95% CI)	Yes	No	AOR (95% CI)
Income 10,001–20,000	112 (60.9%)	72 (39.1%)	1.29 (0.68–2.45)	138 (75.0%)	46 (25.0%)	2.51 (1.07–5.90)*
Nursing	138 (75.0%)	46 (25.0%)	0.46 (0.26–0.81)*	145 (78.8%)	39 (21.2%)	0.81 (0.42–1.57)
Rehabilitation therapist	69 (70.4%)	29 (29.6%)	0.55 (0.30–0.99)*	86 (87.8%)	12 (12.2%)	0.40 (0.18–0.88)*
Having no children	185 (60.9%)	119 (39.1%)	2.14 (1.28–3.57)*	240 (78.9%)	64 (21.1%)	1.55 (0.84–2.87)

AOR, adjusted odds ratio; CI, confidence interval; CNY, Chinese Yuan. *p < 0.05. Reference categories for all variables are specified in the supplementary tables (Supplementary Tables 1–6).

Finally, a significant discrepancy was observed between first-person (for one’s own child) and third-person (for another’s child) perspectives regarding LST limitation (*p* < 0.001, Figure 2C). Chi-square tests indicated that for UWS patients, the proportion of HCPs who would limit LST was significantly lower when considering their own child compared to another’s child (34.0% vs. 52.1%, *p* < 0.001). Similarly, for MCS patients, the proportion who would limit LST was also significantly lower from the first-person perspective (18.7% vs. 31.6%, p < 0.001).

### 3.3 Perspectives on pain in pediatric patients with disorders of consciousness

When investigating healthcare professionals’ attitudes toward the perceived ability to feel pain in pediatric patients with UWS and MCS, Chi-square analysis revealed a statistically significant difference between the two conditions (*χ*^2^ = 57.961, *p* < 0.001), as shown in Figure 2D. 89.9% of participants believed that MCS patients could perceive pain, while 65.1% held the same belief for UWS patients.

After Bonferroni correction, the perception that UWS patients could feel pain was found to vary significantly with the number of children the participant had (*χ*^2^ = 7.353, *p* = 0.025). Binary logistic regression indicated that the number of children was inversely associated with the belief that UWS patients could perceive pain (OR = 0.622, 95% CI: 0.416–0.930, *p* = 0.021).

Further analysis revealed that religious belief (*χ*^2^ = 8.445, *p* = 0.004), specific occupation (*χ*^2^ = 11.394, *p* = 0.044), and number of children (*χ*^2^ = 9.002, *p* = 0.011) influenced participants’ attitudes toward MCS patients’ ability to feel pain. Binary logistic regression results (Table 4) showed that, using neurologists as the reference group, other healthcare professionals were more inclined to believe that MCS patients could perceive pain (AOR = 2.88, 95% CI: 1.11–7.46).

**Table 4:** Beliefs of other healthcare professionals regarding pain perception in pediatric patients with DoC (*n* = 656).

Item	Believes child with UWS can perceive pain?			Believes child with MCS can perceive pain?		
	Yes	No	AOR (95% CI)	Yes	No	AOR (95% CI)
Other healthcare professional	68 (61.8%)	42 (38.2%)	0.96 (0.55–1.68)	93 (84.5%)	17 (15.5%)	2.88 (1.11–7.46)*

AOR, adjusted odds ratio; CI, confidence interval. Using neurologists as the reference group. *p < 0.05. Reference categories for all variables are specified in the supplementary tables (Supplementary Tables 1–6).

We also performed chi-square tests to examine the relationship between healthcare professionals’ decisions to limit LST and their perception of pain in pediatric patients. When participants were making decisions for another’s child (Figure 2E), for UWS patients, the choice to limit LST was statistically associated with pain perception (*χ*^2^ = 4.277, *p* = 0.039). For MCS patients, however, the perception of pain was not significantly associated with the inclination to limit life-sustaining treatment (*χ*^2^ = 1.359, *p* = 0.244). When participants considered the scenario for their own child (Figure 2F), for UWS patients, the perception that the child could feel pain was associated with a lower willingness to limit LST (i.e., greater inclination to continue LST) (*χ*^2^ = 9.854, *p* = 0.002). For MCS patients, pain perception showed no statistically significant effect on the willingness to limit treatment (*χ*^2^ = 1.453, *p* = 0.228).

## 4 Discussion

This study represents the first comprehensive investigation into Chinese healthcare professionals’ attitudes regarding pain perception and life-sustaining treatment decision-making for children with disorders of consciousness (DoC), while also exploring factors influencing the LST decision-making process.

Overall, we found that over half of the respondents preferred limiting LST for a child with unresponsive wakefulness syndrome (UWS) when considering another’s child. However, this preference substantially decreased support when the scenario involved their own child, with 66% opting to continue LST. This stark contrast may reflect the profound tension between professional judgment based on clinical prognosis and emotional considerations driven by kinship, within broader economic and cultural contexts. These findings provide empirical evidence for developing more standardized and nuanced clinical management protocols for pediatric DoC.

The study found significant differences in healthcare professionals’ support for limiting life-sustaining treatment from pediatric patients with disorders of consciousness (DoC) (*p* < 0.001), with higher support for children in an unresponsive wakefulness syndrome (UWS) (52.1%) compared to those in a minimally conscious state (MCS) (31.6%). Furthermore, these rates were significantly lower than those reported for adult DoC patients in China (UWS: 53.3%; MCS: 37.2%) and for adult patients in European studies (UWS: 66%; MCS: 28%) (3, 34, 36). This discrepancy may be explained by several factors, including differences in healthcare professionals’ understanding of pediatric neurophysiology, distinctions between DoC subtypes, and China’s sociocultural and ethical-legal context. Specifically, the natural disease course and prognosis of pediatric DoC remain poorly defined, while the likelihood of recovery for children with UWS diminishes significantly over time (41). This is particularly true for children under the age of two, who tend to have poor functional outcomes following severe brain injury (42). Conversely, a body of research highlights the superior neuroplasticity of the developing brain, which exhibits a much stronger ability to form new neural pathways and compensate for damage compared to the adult brain. It is crucial to interpret this potential within the context of injury severity. While severe injuries in young children still carry a high risk of poor recovery, this enhanced plasticity is considered a key factor in the documented occurrences of consciousness regaining beyond 12 months, observed more frequently in children than in adults with comparable injuries (10, 15). A European cohort study on pediatric/adolescent DoC demonstrated that children in a minimally conscious state (MCS) had a higher likelihood of receiving rehabilitation therapy (86%) compared to those with unresponsive wakefulness syndrome (UWS) (49%), and were associated with better outcomes (16, 43). Given this prognostic uncertainty, structured prognostic counseling should be provided to families of children with DoC. This process is essential to educate families and support their involvement in shared decision-making regarding the overall care plan, which may include decisions about continuing or transitioning away from rehabilitative therapies, with the ultimate goal of improving the child’s quality of life (10). Currently, there are no specific regulations in China regarding the limitation of life-sustaining treatment, and clinical decision-making relies heavily on medical ethical consensus and institutional review procedures (44). To mitigate the risk of medical disputes, healthcare professionals often align their practice with the preferences of the child’s legal guardians. Consequently, in the decision-making process regarding treatment limitation, the primary responsibility and burden frequently fall upon the guardians. However, such decisions may not always align with the best interests of the child, presenting significant ethical concerns and potential risks (44). Furthermore, the Confucian tenet of “sanctity of life,” which views children as essential to familial and bloodline continuity, exerts a significant influence on the decision-making process, representing a deep-seated cultural value (45). The cultural imperative to preserve life, however, operates within a context of practical constraints. Foremost among these is the economic burden on families, which our findings identify as a critical determinant in clinicians’ support for LST limitation (46, 47), our findings also highlight the pivotal role of resource limitations in healthcare decision-making in low- and middle-income countries (48).

This pattern suggests a non-linear association between income and LST decision-making. Specifically, for children with MCS, a state characterized by greater prognostic uncertainty, the middle-income bracket (10,001–20,000 CNY) emerged as a significant predictor of continuing LST when the decision involved one’s own child (AOR = 2.51), whereas high income showed no significant effect (49). This suggests that economic capacity, rather than absolute income level, interacts with prognostic uncertainty and the emotional stakes of kinship (50, 51). In particular, individuals in the middle-income group may have sufficient financial resources to sustain prolonged treatment while remaining strongly influenced by hope for recovery, whereas higher-income individuals may rely more on prognostic evaluation than resource constraints. From a third-person perspective, higher income was associated with a greater likelihood of supporting LST limitation when considering another’s child with UWS, suggesting that economic considerations may play a more prominent role in decision-making when emotional involvement is lower and prognosis is relatively poor. Furthermore, professional roles and personal life experiences significantly modulate these neuroethical decisions through a lens of “clinical realism.” Notably, nursing staff (AOR = 0.46) and rehabilitation therapists (AOR = 0.55) demonstrated a significantly lower willingness to continue LST for their own child with UWS, a trend that persisted for therapists in MCS scenarios (AOR = 0.40) (52, 53). HCPs without children were more likely to continue LST for UWS patients (AOR = 2.14, 95% CI: 1.28–3.57). This suggests that individuals without firsthand parental experience may hold a more idealized “sanctity of life” view, potentially underestimating the cumulative psychosocial and financial strain of raising a child with severe neurological impairment (54, 55). In summary, the decision to maintain life-sustaining treatment is not a purely clinical judgment but a complex balance of familial economic constraints, professional “frontline” exposure, and personal life stages. Optimizing this process necessitates structured educational support to guide ethical deliberation and shared decision-making.

Finally, the majority of healthcare professionals believed that pediatric DoC patients can perceive pain (UWS: 65.1%; MCS: 89.9%), consistent with findings in adult studies (UWS: 59%; MCS: 96%) (56). These views are likely based on functional neuroimaging data and clinical experience, which acknowledge a potential covert capacity for subjective pain even in non-communicative states (57). Views on pain perception varied significantly across specialties (MCS: *p* = 0.044) and correlated with parental status (UWS: *p* = 0.025; MCS: *p* = 0.010), potentially reflecting disparate inter-professional pain cognition frameworks (58). Furthermore, the emotional bond inherent in parenthood may enable caregivers to detect subtle behavioral cues of pain more keenly, a factor crucial for developing pediatric-specific care protocols (59, 60). The influence of pain perception on LST decisions exhibited a diagnostic-specific pattern in our study. For patients in UWS, the perception that a child could experience pain was associated with a significantly stronger willingness to continue LST (*p* < 0.05), particularly from a first-person perspective. In this context, the capacity for pain may be interpreted as a surrogate indicator, providing a moral and clinical rationale to maintain life-sustaining efforts (61).

For MCS patients, pain perception did not significantly influence LST decisions. Given that nearly 90% of respondents acknowledged pain capacity in MCS, such perception no longer functions as a diagnostic differentiator for treatment limitation. Instead, it serves as a universal clinical mandate for enhanced analgesic management and compassionate care, regardless of the eventual LST decision. This underscores the necessity of incorporating standardized pain ethics into the clinical reasoning framework for pediatric DoC management (62).

It is important to note that the association between childlessness and a greater willingness to continue LST for a child with UWS should be interpreted with caution. Given the cross-sectional design of this study, causality cannot be inferred, and the observed difference may reflect unmeasured confounding factors. For instance, healthcare professionals without children may differ systematically from parents in age, clinical seniority, or cultural exposure, all of which could independently influence end-of-life decision-making. Furthermore, the childlessness variable itself is heterogeneous, encompassing individuals who have chosen to remain child-free as well as those who plan to have children in the future, and the underlying motivations and psychological profiles of these subgroups may diverge. Thus, while the finding aligns with the hypothesis that personal parenting experience tempers idealized views of life-sustaining treatment, it should be regarded as hypothesis-generating rather than definitive. Future qualitative or longitudinal research is warranted to disentangle the complex interplay between personal life history and clinical ethical judgment.

## 5 Strengths and limitations

This study represents the first comprehensive investigation into the attitudes of Chinese healthcare professionals regarding pain perception and life-sustaining treatment decision-making for pediatric patients with disorders of consciousness (DoC), thereby addressing a critical gap in the literature. The study’s primary strengths are threefold. First, it focuses on a unique population, pediatric DoC patients, who present distinct challenges in both neuroethics and clinical practice; the findings offer valuable insights for optimizing care for the next generation of critically ill children. Second, the research builds upon a solid methodological foundation: the questionnaire was adapted from a well-established tool developed by the internationally recognized Coma Science Group, undergoing forward-backward translation, expert panel review, and pretesting to ensure semantic, conceptual, and cultural equivalence. Third, through a multi-center strategy, we collected responses from 656 healthcare professionals across eight Chinese provinces and municipalities, encompassing diverse professional backgrounds, thereby achieving a robust sample size for a survey study and providing substantial support for the conclusions. Notably, although nurses and rehabilitation therapists are not the final decision-makers in LST limitation in China, their inclusion in this study is justified by their integral role in the multidisciplinary care team, capturing the full spectrum of attitudes that influence LST decisions in practice.

However, several limitations should be acknowledged. First, the cross-sectional design captures attitudes at a single point in time and cannot track how decision preferences might evolve with clinical experience, policy changes, or personal circumstances. Second, despite the multi-center design, the sample may still be subject to sampling bias. The participants were recruited primarily from tertiary hospitals in specific regions of China, which may not fully represent healthcare professionals in community hospitals or less-developed areas. Additionally, the use of convenience sampling and voluntary response could have led to an overrepresentation of individuals with stronger opinions or greater interest in pediatric DoC, potentially skewing the results. This limits the generalizability of our findings to the broader population of healthcare professionals across different clinical settings and cultural contexts within China. Third, although the study covers the main DoC subtypes (UWS and MCS), it did not further distinguish within the minimally conscious state between MCS+ (high-level behavioral responses) and MCS− (low-level responses), despite potential prognostic differences that could influence clinical decision-making. Taken together, these findings support a tripartite decision framework integrating prognosis, emotional proximity, and economic capacity. Future research should employ longitudinal designs and incorporate more refined behavioral or neuroimaging diagnostic classifications to better elucidate the trajectory of attitude formation and its underlying neurobiological mechanisms.

## 6 Conclusion

This study reveals significant variations in Chinese HCPs’ LST decisions for pediatric DoC, shaped by the child’s diagnosis, the decision-making perspective, and perceptions of pain. HCPs are generally more reluctant to limit LST for children compared to adults, particularly when considering their own offspring. Key determinants include professional role, parental status, and a context-dependent economic capacity.

The gap between third-person professional judgment and first-person emotional kinship illustrates an “ethical transposition” in pediatric DoC care. Clinical decision-making for these vulnerable patients must transcend purely prognostic metrics, integrating the lived experiences of front-line caregivers and the socioeconomic realities of the family. To safeguard the patient’s best interests, future frameworks should adopt a more patient- and family-centered ethical framework that reconciles clinical realism with the culturally deep-seated values of kinship and family integrity.

## Data Availability

The original contributions presented in the study are included in the article/Supplementary material, further inquiries can be directed to the corresponding authors.
