# Supplementary material for: Pain perception and attitudes toward life-sustaining treatment in pediatric patients with disorders of consciousness: a survey of Chinese healthcare professionals
Source: Front Neurol. 2026 Jul 6;17:1765041. doi: 10.3389/fneur.2026.1765041 (PMC13381256; doi:10.3389/fneur.2026.1765041)
Supplement: Supplementary file 1 [file Data_Sheet_1.DOCX]

**Supplementary Table 1. Binary Logistic Regression Analysis of Factors Associated with HCPs’ Attitudes Toward Limiting LST for Another’s Child with UWS (n=656)**

| Item | Category | B | Standard Error | Wald | df | P value | OR (95% CI) |
| --- | --- | --- | --- | --- | --- | --- | --- |
| **Age** | 20-30 years (ref) |  |  |  |  |  |  |
|  | 31-40 years | 0.042 | 0.243 | 0.030 | 1 | 0.863 | 1.043 (0.648-1.679) |
|  | 41-50 years | 0.104 | 0.346 | 0.090 | 1 | 0.764 | 1.109 (0.563-2.184) |
|  | >50 years | 0.450 | 0.475 | 0.898 | 1 | 0.343 | 1.568 (0.618-3.974) |
| **Gender** | Male (ref) |  |  |  |  |  |  |
|  | Female | 0.207 | 0.180 | 1.319 | 1 | 0.251 | 1.229 (0.864-1.749) |
| **Religious belief** | Yes (ref) |  |  |  |  |  |  |
|  | No | -0.183 | 0.350 | 0.274 | 1 | 0.600 | 0.833 (0.420-1.652) |
| **Monthly income (CNY)** | ≤2,000 (ref) |  |  |  |  |  |  |
|  | 2,001-5,000 | 0.504 | 0.361 | 1.949 | 1 | 0.163 | 1.656 (0.816-3.361) |
|  | 5,001-10,000 | 0.538 | 0.309 | 3.037 | 1 | 0.081 | 1.713 (0.935-3.137) |
|  | 10,001-20,000 | 0.253 | 0.328 | 0.596 | 1 | 0.440 | 1.288 (0.677-2.450) |
|  | >20,000 | 1.053 | 0.447 | 5.550 | 1 | **0.018** | 2.865 (1.193-6.878) |
| **Education level** | Bachelor’s degree (ref) |  |  |  |  |  |  |
|  | Master’s degree | -0.195 | 0.188 | 1.068 | 1 | 0.301 | 0.823 (0.569-1.191) |
| **Professional category** | Neurologist (ref) |  |  |  |  |  |  |
|  | Other Clinical Doctor | 0.186 | 0.257 | 0.526 | 1 | 0.468 | 1.205 (0.728-1.995) |
|  | Nursing Staff | 0.419 | 0.274 | 2.327 | 1 | 0.127 | 1.520 (0.888-2.602) |
|  | Rehabilitati-on Therapist | 0.471 | 0.287 | 2.695 | 1 | 0.101 | 1.601 (0.913-2.810) |
|  | Other Healthcare Professionals | 0.327 | 0.282 | 1.341 | 1 | 0.247 | 1.386 (0.798-2.410) |
| **Having children** | Yes (ref) |  |  |  |  |  |  |
|  | No | 0.063 | 0.241 | 0.068 | 1 | 0.794 | 1.065 (0.664-1.708) |
| **Constant** |  | -0.755 | 0.533 | 2.006 | 1 | 0.157 | 0.470 |

**Supplementary Table 2. Binary Logistic Regression Analysis of Factors Associated with HCPs’ Attitudes Toward Limiting LST for Another’s Child with MCS (n=656)**

| Item | Category | B | Standard Error | Wald | df | P value | OR (95% CI) |
| --- | --- | --- | --- | --- | --- | --- | --- |
| **Age** | 20-30 years (ref) |  |  |  |  |  |  |
|  | 31-40 years | 0.089 | 0.263 | 0.115 | 1 | 0.734 | 1.094 (0.653-1.832) |
|  | 41-50 years | 0.042 | 0.367 | 0.013 | 1 | 0.909 | 1.043 (0.508-2.142) |
|  | >50 years | 0.653 | 0.538 | 1.470 | 1 | 0.225 | 1.921 (0.669-5.516) |
| **Gender** | Male (ref) |  |  |  |  |  |  |
|  | Female | 0.327 | 0.192 | 2.917 | 1 | 0.088 | 1.387 (0.953-2.019) |
| **Religious belief** | Yes (ref) |  |  |  |  |  |  |
|  | No | -0.100 | 0.385 | 0.068 | 1 | 0.795 | 0.905 (0.426-1.923) |
| **Monthly income (CNY)** | ≤2,000 (ref) |  |  |  |  |  |  |
|  | 2,001-5,000 | -0.582 | 0.389 | 2.237 | 1 | 0.135 | 0.559 (0.260-1.198) |
|  | 5,001-10,000 | 0.005 | 0.345 | 0.000 | 1 | 0.988 | 1.005 (0.511-1.975) |
|  | 10,001-20,000 | -0.608 | 0.358 | 2.887 | 1 | 0.089 | 0.545 (0.270-1.098) |
|  | >20,000 | -0.317 | 0.484 | 0.427 | 1 | 0.513 | 0.729 (0.282-1.882) |
| **Education level** | Bachelor’s degree (ref) |  |  |  |  |  |  |
|  | Master’s degree | 0.306 | 0.205 | 2.222 | 1 | 0.136 | 1.358 (0.908-2.031) |
| **Professional category** | Neurologist (ref) |  |  |  |  |  |  |
|  | Other Clinical Doctor | -0.242 | 0.273 | 0.785 | 1 | 0.376 | 0.785 (0.460-1.340) |
|  | Nursing Staff | -0.279 | 0.296 | 0.891 | 1 | 0.345 | 0.756 (0.424-1.350) |
|  | Rehabilitati-on Therapist | 0.253 | 0.320 | 0.625 | 1 | 0.429 | 1.288 (0.687–2.414) |
|  | Other Healthcare Professionals | –0.007 | 0.306 | 0.000 | 1 | 0.983 | 0.993 (0.545–1.811) |
| **Having children** | Yes (ref) |  |  |  |  |  |  |
|  | No | 0.087 | 0.262 | 0.110 | 1 | 0.740 | 1.091 (0.653-1.823) |
| **Constant** |  | 0.782 | 0.583 | 1.798 | 1 | 0.180 | 2.187 |

**Supplementary Table 3. Binary Logistic Regression Analysis of Factors Associated with HCPs’ Attitudes Toward Continuing LST for Their Own Child with UWS (n=656)**

| Item | Category | B | Standard Error | Wald | df | P value | OR (95% CI) |
| --- | --- | --- | --- | --- | --- | --- | --- |
| **Age** | 20-30 years (ref) |  |  |  |  |  |  |
|  | 31-40 years | 0.110 | 0.262 | 0.176 | 1 | 0.675 | 1.116 (0.668-1.865) |
|  | 41-50 years | 0.072 | 0.378 | 0.036 | 1 | 0.850 | 1.074 (0.512-2.253) |
|  | >50 years | 0.943 | 0.485 | 3.772 | 1 | 0.052 | 2.567 (0.991-6.647) |
| **Gender** | Male (ref) |  |  |  |  |  |  |
|  | Female | -0.066 | 0.188 | 0.124 | 1 | 0.724 | 0.936 (0.647-1.353) |
| **Religious belief** | Yes (ref) |  |  |  |  |  |  |
|  | No | 0.099 | 0.386 | 0.066 | 1 | 0.798 | 1.104 (0.518-2.353) |
| **Monthly income (CNY)** | ≤2,000 (ref) |  |  |  |  |  |  |
|  | 2,001-5,000 | -0.110 | 0.370 | 0.088 | 1 | 0.766 | 0.896 (0.433-1.851) |
|  | 5,001-10,000 | -0.176 | 0.313 | 0.318 | 1 | 0.573 | 0.838 (0.454-1.547) |
|  | 10,001-20,000 | 0.163 | 0.331 | 0.243 | 1 | 0.622 | 1.177 (0.615-2.254) |
|  | >20,000 | -0.112 | 0.459 | 0.060 | 1 | 0.807 | 0.894(0.363-2.200 |
| **Education level** | Bachelor’s degree (ref) |  |  |  |  |  |  |
|  | Master’s degree | 0.171 | 0.198 | 0.746 | 1 | 0.388 | 1.186 (0.805-1.748) |
| **Professional category** | Neurologist (ref) |  |  |  |  |  |  |
|  | Other Clinical Doctor | -0.265 | 0.261 | 1.030 | 1 | 0.310 | 0.768 (0.461-1.279) |
|  | Nursing Staff | -0.786 | 0.293 | 7.183 | 1 | **0.007** | 0.456 (0.257-0.810) |
|  | Rehabilitation Therapist | -0.606 | 0.304 | 3.973 | 1 | **0.046** | 0.546 (0.301-0.990) |
|  | Other Healthcare Professionals | -0.334 | 0.290 | 1.326 | 1 | 0.249 | 0.716 (0.406-1.264) |
| **Having children** | Yes (ref) |  |  |  |  |  |  |
|  | No | 0.761 | 0.261 | 8.489 | 1 | **0.004** | 2.141 (1.283-3.574) |
| **Constant** |  | -0.803 | 0.564 | 2.028 | 1 | 0.154 | 0.448 |

**Supplementary Table 4. Binary Logistic Regression Analysis of Factors Associated with HCPs’ Attitudes Toward Continuing LST for Their Own Child with MCS (n=656)**

| Item | Category | B | Standard Error | Wald | df | P value | OR (95% CI) |
| --- | --- | --- | --- | --- | --- | --- | --- |
| **Age** | 20-30 years (ref) |  |  |  |  |  |  |
|  | 31-40 years | -0.066 | 0.312 | 0.045 | 1 | 0.833 | 0.936 (0.508-1.725) |
|  | 41-50 years | -0.143 | 0.450 | 0.100 | 1 | 0.751 | 0.867 (0.359-2.096) |
|  | >50 years | -0.442 | 0.623 | 0.504 | 1 | 0.478 | 0.643 (0.190-2.178) |
| **Gender** | Male (ref) |  |  |  |  |  |  |
|  | Female | -0.121 | 0.230 | 0.278 | 1 | 0.598 | 0.886 (0.564-1.390) |
| **Religious belief** | Yes (ref) |  |  |  |  |  |  |
|  | No | 0.328 | 0.509 | 0.416 | 1 | 0.519 | 1.389 (0.512-3.767) |
| **Monthly income (CNY)** | ≤2,000 (ref) |  |  |  |  |  |  |
|  | 2,001-5,000 | 0.603 | 0.470 | 1.643 | 1 | 0.200 | 1.827 (0.727-4.594) |
|  | 5,001-10,000 | 0.035 | 0.428 | 0.007 | 1 | 0.936 | 1.035 (0.448-2.394) |
|  | 10,001-20,000 | 0.921 | 0.435 | 4.484 | 1 | **0.034** | 2.513 (1.071-5.895) |
|  | >20,000 | 0.831 | 0.577 | 2.072 | 1 | 0.150 | 2.295(0.741-7.112 |
| **Education level** | Bachelor’s degree (ref) |  |  |  |  |  |  |
|  | Master’s degree | -0.366 | 0.246 | 2.208 | 1 | 0.137 | 0.693 (0.428-1.124) |
| **Professional category** | Neurologist (ref) |  |  |  |  |  |  |
|  | Other Clinical Doctor | -0.310 | 0.315 | 0.968 | 1 | 0.325 | 0.733 (0.395-1.361) |
|  | Nursing Staff | -0.214 | 0.339 | 0.399 | 1 | 0.528 | 0.807 (0.415-1.569) |
|  | Rehabilitation Therapist | -0.914 | 0.398 | 5.261 | 1 | **0.022** | 0.401 (0.184-0.875) |
|  | Other Healthcare Professionals | -0.552 | 0.365 | 2.284 | 1 | 0.131 | 0.576 (0.281-1.178) |
| **Having children** | Yes (ref) |  |  |  |  |  |  |
|  | No | 0.440 | 0.312 | 1.988 | 1 | 0.159 | 1.553 (0.842-2.866) |
| **Constant** |  | -1.802 | 0.737 | 5.972 | 1 | 0.015 | 0.165 |

**Supplementary Table 5. Binary Logistic Regression Analysis of Factors Associated with HCPs’ Attitudes Toward Limiting LST for Their Own Child with UWS (n=656)**

| Item | Category | B | Standard Error | Wald | df | P value | OR (95% CI) |
| --- | --- | --- | --- | --- | --- | --- | --- |
| **Age** | 20-30 years (ref) |  |  |  |  |  |  |
|  | 31-40 years | 0.141 | 0.251 | 0.317 | 1 | 0.574 | 1.152 (0.704-1.885) |
|  | 41-50 years | 0.077 | 0.362 | 0.045 | 1 | 0.832 | 1.080 (0.531-2.193) |
|  | >50 years | -0.196 | 0.512 | 0.146 | 1 | 0.702 | 0.822 (0.302-2.242) |
| **Gender** | Male (ref) |  |  |  |  |  |  |
|  | Female | 0.175 | 0.187 | 0.879 | 1 | 0.348 | 1.192 (0.826-1.719) |
| **Religious belief** | Yes (ref) |  |  |  |  |  |  |
|  | No | 0.101 | 0.365 | 0.077 | 1 | 0.781 | 1.107 (0.541-2.262) |
| **Monthly income (CNY)** | ≤2,000 (ref) |  |  |  |  |  |  |
|  | 2,001-5,000 | -0.245 | 0.367 | 0.444 | 1 | 0.505 | 0.783 (0.381-1.608) |
|  | 5,001-10,000 | -0.121 | 0.309 | 0.152 | 1 | 0.696 | 0.886 (0.484-1.625) |
|  | 10,001-20,000 | -0.169 | 0.330 | 0.263 | 1 | 0.608 | 0.845 (0.443-1.611) |
|  | >20,000 | -0.034 | 0.456 | 0.006 | 1 | 0.941 | 0.967(0.396-2.361) |
| **Education level** | Bachelor’s degree (ref) |  |  |  |  |  |  |
|  | Master’s degree | -0.171 | 0.196 | 0.755 | 1 | 0.385 | 0.843 (0.574-1.239) |
| **Professional category** | Neurologist (ref) |  |  |  |  |  |  |
|  | Other Clinical Doctor | -0.086 | 0.263 | 0.107 | 1 | 0.744 | 0.918 (0.548-1.537) |
|  | Nursing Staff | -0.315 | 0.282 | 1.251 | 1 | 0.263 | 0.730 (0.420-1.268) |
|  | Rehabilitation Therapist | -0.451 | 0.302 | 2.223 | 1 | 0.136 | 0.637 (0.352–1.152) |
|  | Other Healthcare Professionals | -0.041 | 0.286 | 0.020 | 1 | 0.887 | 0.960 (0.548-1.682) |
| **Having children** | Yes (ref) |  |  |  |  |  |  |
|  | No | 0.340 | 0.249 | 1.856 | 1 | 0.173 | 1.405 (0.862-2.290) |
| **Constant** |  | -0.670 | 0.546 | 1.505 | 1 | 0.220 | 0.512 |

**Supplementary Table 6. Binary Logistic Regression Analysis of Factors Associated with HCPs’ Attitudes Toward Limiting LST for Their Own Child with MCS (n=656)**

| Item | Category | B | Standard Error | Wald | df | P value | OR (95% CI) |
| --- | --- | --- | --- | --- | --- | --- | --- |
| **Age** | 20-30 years (ref) |  |  |  |  |  |  |
|  | 31-40 years | 0.228 | 0.396 | 0.331 | 1 | 0.565 | 1.256 (0.578-2.732) |
|  | 41-50 years | -0.114 | 0.600 | 0.036 | 1 | 0.849 | 0.892 (0.275-2.892) |
|  | >50 years | -0.095 | 0.879 | 0.012 | 1 | 0.914 | 0.910 (0.162-5.097) |
| **Gender** | Male (ref) |  |  |  |  |  |  |
|  | Female | -0.010 | 0.305 | 0.001 | 1 | 0.973 | 0.990 (0.544-1.799) |
| **Religious belief** | Yes (ref) |  |  |  |  |  |  |
|  | No | 0.937 | 0.758 | 1.531 | 1 | 0.216 | 2.553 (0.578-11.271) |
| **Monthly income (CNY)** | ≤2,000 (ref) |  |  |  |  |  |  |
|  | 2,001-5,000 | -0.192 | 0.536 | 0.128 | 1 | 0.721 | 0.826 (0.289-2.360) |
|  | 5,001-10,000 | -0.691 | 0.477 | 2.098 | 1 | 0.147 | 0.501 (0.197-1.276) |
|  | 10,001-20,000 | -0.291 | 0.498 | 0.342 | 1 | 0.559 | 0.747 (0.282-1.982) |
|  | >20,000 | -0.128 | 0.701 | 0.033 | 1 | 0.855 | 0.880(0.223-3.474 |
| **Education level** | Bachelor’s degree (ref) |  |  |  |  |  |  |
|  | Master’s degree | -0.194 | 0.320 | 0.367 | 1 | 0.545 | 0.824 (0.440-1.543) |
| **Professional category** | Neurologist (ref) |  |  |  |  |  |  |
|  | Other Clinical Doctor | 0.529 | 0.482 | 1.207 | 1 | 0.272 | 1.698 (0.660-4.367) |
|  | Nursing Staff | 0.889 | 0.497 | 3.199 | 1 | 0.074 | 2.432 (0.918-6.442) |
|  | Rehabilitation Therapist | -0.183 | 0.611 | 0.090 | 1 | 0.764 | 0.833 (0.251-2.757) |
|  | Other Healthcare Professionals | 1.057 | 0.486 | 4.730 | 1 | **0.030** | 2.878 (1.110-7.461) |
| **Having children** | Yes (ref) |  |  |  |  |  |  |
|  | No | -0.112 | 0.395 | 0.081 | 1 | 0.776 | 0.894 (0.412-1.937) |
| **Constant** |  | -3.206 | 1.019 | 9.907 | 1 | 0.002 | 0.041 |
